# A Vision-Aided 3D Path Teaching Method before Narrow Butt Joint Welding

**DOI:** 10.3390/s17051099

**Published:** 2017-05-11

**Authors:** Jinle Zeng, Baohua Chang, Dong Du, Guodong Peng, Shuhe Chang, Yuxiang Hong, Li Wang, Jiguo Shan

**Affiliations:** Key Laboratory for Advanced Materials Processing Technology, Ministry of Education, Department of Mechanical Engineering, Tsinghua University, Beijing 100084, China; zengjl12@mails.tsinghua.edu.cn (J.Z.); bhchang@tsinghua.edu.cn (B.C.); pengguodong12@163.com (G.P.); changsh15@mails.tsinghua.edu.cn (S.C.); hongyuxiang@tsinghua.edu.cn (Y.H.); wanglidme@tsinghua.edu.cn (L.W.); shanjg@tsinghua.edu.cn (J.S.)

**Keywords:** seam tracking, visual detection, narrow butt joint recognition, position and pose detection, automatic path teaching

## Abstract

For better welding quality, accurate path teaching for actuators must be achieved before welding. Due to machining errors, assembly errors, deformations, etc., the actual groove position may be different from the predetermined path. Therefore, it is significant to recognize the actual groove position using machine vision methods and perform an accurate path teaching process. However, during the teaching process of a narrow butt joint, the existing machine vision methods may fail because of poor adaptability, low resolution, and lack of 3D information. This paper proposes a 3D path teaching method for narrow butt joint welding. This method obtains two kinds of visual information nearly at the same time, namely 2D pixel coordinates of the groove in uniform lighting condition and 3D point cloud data of the workpiece surface in cross-line laser lighting condition. The 3D position and pose between the welding torch and groove can be calculated after information fusion. The image resolution can reach 12.5 μm. Experiments are carried out at an actuator speed of 2300 mm/min and groove width of less than 0.1 mm. The results show that this method is suitable for groove recognition before narrow butt joint welding and can be applied in path teaching fields of 3D complex components.

## 1. Introduction

In 3D complex component welding fields, the relative motion path between the welding torch and the workpiece must be reasonably planned beforehand to achieve good weld quality, so that the welding torch is keep aligned with the groove during the whole welding process [1,2,3,4,5,6]. The recognition of the actual groove position is a prerequisite for motion planning before welding. Currently, the “teaching-playback” mode is widely used in most automatic actuators such as welding robots [7,8,9]. The workers recognize the groove position by their eyes and guide the actuators to move along the groove. The actuators record the groove position during guidance so that they can move along the recorded path in the following welding process. The “teaching-playback” mode is usually rather time-consuming and not accurate enough. Many robot manufacturers have developed some offline programming software [10,11,12,13] that can perform automatic path planning by computers based on the component’s CAD model. However, machining errors, assembly errors, deformations, etc. may cause deviations between the actual groove position and the path predetermined by the “teaching-playback” or the offline programming method. The “teaching-playback” mode and offline programming technique cannot correct the deviations caused by the factors above. Therefore, it is essential to develop the automatic groove recognition method to detect the 3D position of the actual groove.

**Figure 1:**
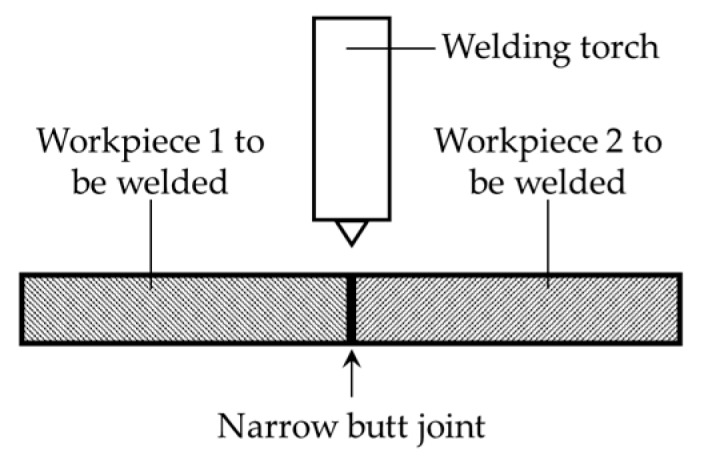
The cross section of the narrow butt joint. The workpieces to be welded are closely assembled and no gap is reserved purposely.

Compared with other groove recognition methods (arc sensing [14], tactile sensing [15], acoustic sensing [16], eddy current sensing [17], etc.), the machine vision method does not need to contact the workpiece and usually gives more abundant information and more accurate results. It has been considered to be one of the most promising groove recognition technologies [18]. However, there are still many serious challenges when detecting the groove position of aerospace and precision instrument components. First, for precise and effective welding without filling too many metals, the components to be welded are usually so closely assembled that no gap is reserved purposely, which is called the “narrow butt joint.” The cross section of the narrow butt joint is shown in Figure 1. The width of the narrow butt joint may be less than 0.1 mm, and it is necessary to develop a precise detection method in this condition. Second, the camera only captures 2D images of the workpiece surface. However, in 3D groove welding fields, both the 3D position and normal vector coordinates of each point in the groove curve must be obtained.

**Figure 2:**
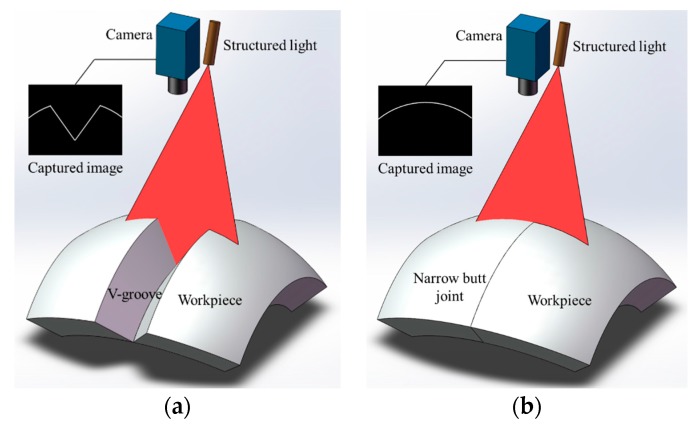
The structured light detection method applied in V-groove and narrow butt joint detection. (**a**) V-groove detection; (**b**) Narrow butt joint detection.

The structured light detection method is currently the most widely used groove recognition technique in industries. Many companies such as Meta Vision [19], Servo Robot [20], Scout [21], and Scansonic [22] have developed various structured light sensors. These sensors project laser stripes onto the workpiece surface and the stripes become distorted due to the geometric differences between the base metal and the groove [23,24,25,26,27]. The groove position is then recognized by detecting the inflection points of the laser stripe curves. Moreover, the 3D cross-section information of the groove can be obtained based on the triangulation principle. The structured light detection method has proved its applicability when there are significant geometric differences between the base metal and the groove, as shown in Figure 2a. However, it may fail in narrow butt joint detection because of the nearly zero gap between the workpieces, as shown in Figure 2b.

Researchers have proposed many other methods to achieve accurate narrow butt joint detection. Shen [28], Chen [29], and Ma [30] captured the images of narrow butt joint in positive lighting conditions (auxiliary light source needed) or passive lighting conditions (no auxiliary light source needed). The position of the narrow butt joint is recognized based on the grayscale gradient differences between the base metal and the groove. Huang used a laser stripe with a large width and projected it onto the workpiece surface [31]. The gap of the narrow butt joint would result in the discontinuity of the laser stripe curve, which indicates the groove position. These methods mentioned above can only obtain the 2D groove position using the grayscale image of the workpiece surface, but the distance and angles between the welding torch and the workpiece surface are necessary in 3D groove welding.

There are also several 3D groove detection methods proposed by other researchers. Zheng proposed a 3D groove detection method using a circular laser spot projected onto the workpiece surface [32,33]. The projected laser spot on the workpiece surface was deformed and a camera was used to capture images of the elliptical laser spot. The angle between the workpiece surface and the camera was calculated using the major axis length and minor axis length of the spot. In addition, the position and direction deviation between the groove and the welding torch was directly represented by the position and direction of the groove curve in the image. Zheng’s calculation method above implies an assumption that the camera captured images based on parallel projection principle, which is inaccurate because most of the cameras captured image based on the perspective projection principle. Zeng proposed another 3D groove detection method combining various visual information in different lighting conditions [18]. This method only gives the solution when the workpiece surface is approximately a flat plane. Moreover, no 3D groove detection experiment is shown in this method. In summary, there are no suitable 3D groove detection method in narrow butt joint recognition fields.

This paper proposes a 3D groove detection method to solve the problems in narrow butt joint recognition fields, which can be used in automatic path teaching before welding. This method captures images when an LED surface light source and a cross-line laser light source are on. The 2D pixel coordinates of the groove curve can be calculated when LED surface light source is on. Meanwhile, we can obtain the 3D point cloud of the workpiece surface when cross-line laser light source is on. After fusing these two kinds of information, we can calculate the 3D position and normal vector coordinates of each point in the groove curve. The LED surface light source and cross-line laser light source are both controlled by the trigger signal, which makes two light sources switch on alternately. The camera is synchronized to capture images when each light source is on. In this way, we can detect 3D position and pose between the welding torch and the workpiece nearly at the same time. Finally, 3D groove detection experiments are carried out to verify the proposed method. Calibration is needed before the system works: the intrinsic parameters of the camera are calibrated using Zhang’s method [34], the light plane equations of the cross-line laser in camera coordinate system are calibrated using Zou’s method [35], and the rotational and translational transformation matrices between camera coordinate system and world coordinate system are calibrated using a common hand-eye calibration method.

The rest of the paper is organized as follows: Section 2 introduces the configuration of our experiment platform and visual sensor. Section 3 illustrates the synchronization method of the camera, LED surface light source and cross-line laser light source. We produce trigger signal at the end of each exposure. Section 4 proposes the processing algorithm when LED surface light source is on. The pixel coordinate of each point in the groove curve would be extracted after image processing. Section 5 proposes the processing algorithm when cross-line laser light source is on. We obtain the 3D point cloud data of the workpiece surface. Combining the pixel coordinate of the groove curve and 3D point cloud of the workpiece surface, we calculate the 3D position and normal vector coordinates of the groove. 3D groove recognition experiments are carried out in Section 6, and conclusions are given in Section 7.

## 2. Configuration of the Experiment Platform

**Figure 3:**
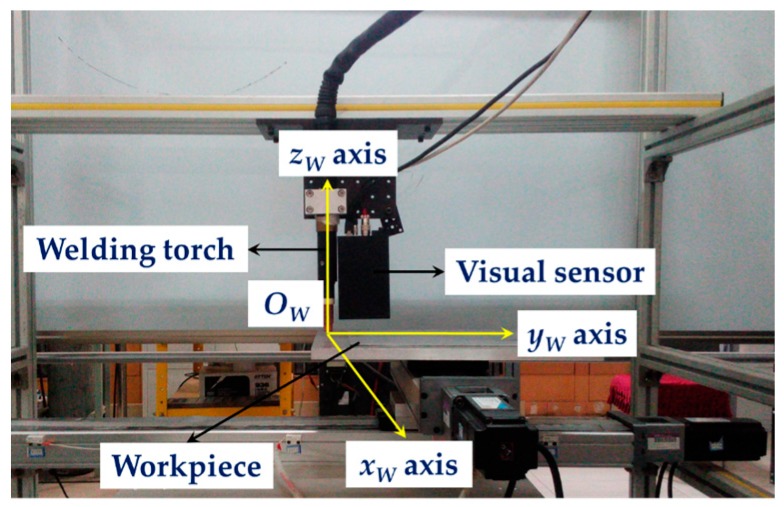
The experiment platform and the world coordinate system used in this paper.

The experiment platform used in this paper is shown in Figure 3. It is comprised of a GTAW welding torch, a visual sensor, a 2D translational stage and an industrial computer. The visual sensor is mounted in front of the welding torch. The world coordinate system {*W*} is fixed on the welding torch, in which the *x_W_* axis and *y_W_* axis of {*W*} are parallel with two moving directions of the 2D translational stage respectively. The workpiece is placed on the terminal of the 2D translational stage, so that it can move along the *x_W_* axis and *y_W_* axis with the translational stage. The CPU frequency of the industrial computer is 2.30 GHz and the RAM size is 4 GB.

When the workpiece moves with the translational stage, the visual sensor detects the position of the narrow butt joint. Meanwhile, the industrial computer processes the image captured by the sensor and calculates the deviations between the groove and the FOV (field of view) of the sensor. Then the industrial computer controls the movement of the workpiece, so that the narrow butt joint always locates in the FOV of the sensor. In this way, the whole groove curve can be recognized and the path teaching process is finished.

**Figure 4:**
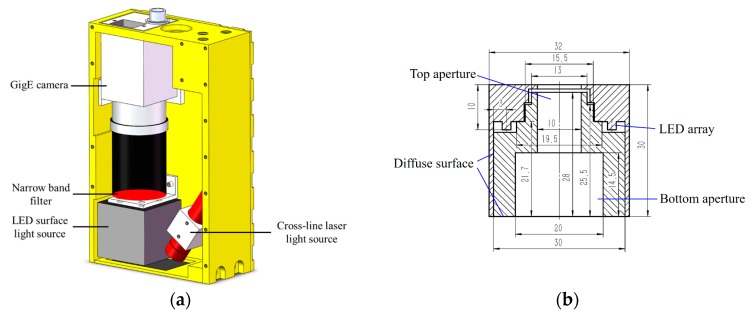
The configuration of our visual sensor and LED surface light source (LSLS) for narrow butt joint detection. (**a**) The configuration of the visual sensor; (**b**) The configuration of the LSLS. The unit of the dimensions in the figure is millimeter (mm).

The configuration of our visual sensor is shown in Figure 4a. It is comprised of a Gigabit Ethernet (GigE) camera, a narrow band filter, a LED surface light source (LSLS), and a cross-line laser light source (CLLLS). The GigE camera is Basler acA1600-60gm, whose maximum frame rate is 60 fps at maximum image size (1200 × 1600 pixels). The power of the LSLS is 6.1 W and the central wavelength is about 630 nm. The wavelength range of the LSLS is about 610 nm to 650 nm. Figure 4b shows the configuration of the LSLS. The light emitted from the LED array is reflected by the diffuse surfaces, then passes through the bottom aperture, and finally projects onto the workpiece surface. The reflected light from the workpiece surface passes through the bottom aperture and top aperture sequentially and is finally collected by the camera. The power of the CLLLS is 20 mW and the central wavelength is 635 nm. The narrow band filter is used to filter out the ambient light, and its central wavelength and FWHM (full width at half maximum) are 635 nm and 10 nm respectively. The light emitted from LSLS and CLLLS can pass through the narrow band filter. The working distance of our visual sensor is 20 mm to 40 mm, and the FOV is about 15 mm × 20 mm at 30 mm in distance. When the working distance is 30 mm, the image resolution of our visual sensor reaches about 12.5 μm, which is suitable for narrow butt joint detection with extremely small width.

The LSLS and CLLLS are designed to provide different visual information from the workpiece surface. When the LSLS is on, the camera captures the grayscale image of the workpiece surface in uniform lighting condition, by which we obtain the 2D pixel coordinates of the narrow butt joint after image processing. When the CLLLS is on, the camera captures the cross-line laser stripe and we can calculate the 3D coordinate of each point in each stripe when considering the intrinsic parameters of the camera. Therefore, the 3D point cloud data of the workpiece surface can be obtained. Fusing these two kinds of visual information, we can calculate the 3D position and normal vector coordinates of the groove.

## 3. Multiple Visual Features Synchronous Acquisition Method

We design a synchronous acquisition method to obtain two kinds of visual information in different lighting conditions. The LSLS and CLLLS are controlled by the trigger signal, so that they are switched on alternately. The camera is then synchronized to capture images when each light source is on. Traditionally, different information would be obtained using different cameras. However, we only use one camera to capture multiple visual features in our proposed method, which would eliminate the error source from calibration between two separate measurements.

**Figure 5:**
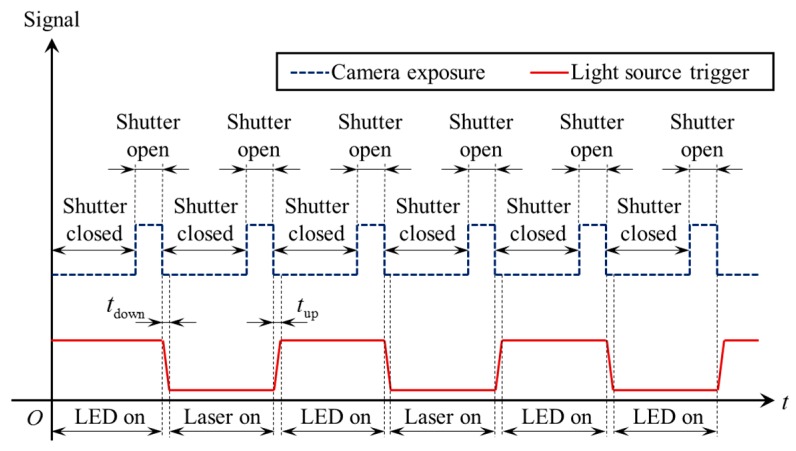
The timing diagrams of the camera exposure signal and light source trigger signal.

Figure 5 shows the timing diagrams of the camera exposure signal and trigger signal. The camera works in continuous acquisition mode, which means that the camera continuously acquires images using its internal clock signal. The camera exposure signal in Figure 5 shows the internal clock signal. The camera starts to capture a new image when the rising edge of the camera exposure signal arrives and finishes capturing when the falling edge comes.

The trigger signal is generated from the digital output port of the camera. The trigger signal stays at high level at the beginning and is automatically inverted by software program at the end of each exposure. The LSLS and CLLLS are respectively switched on during the high level and low level period. Since the trigger signal is inverted at the end of each exposure, it always synchronizes with the camera shutter and its frequency is exactly half of the frame rate. Although the LSLS and CLLLS may be both switched on in the moment the trigger signal is inverted, the exposure of previous frame has stopped and the exposure of next frame does not begin yet (the rising time *t*_up_ and falling time *t*_down_ are both less than 15 μs, which are less than the blanking time between adjacent frames). Therefore, two light sources would not be both switched on in the same exposure period. In this way, different visual information will not be interfered by each other.

## 4. 2D Narrow Butt Joint Recognition Method

**Figure 6:**
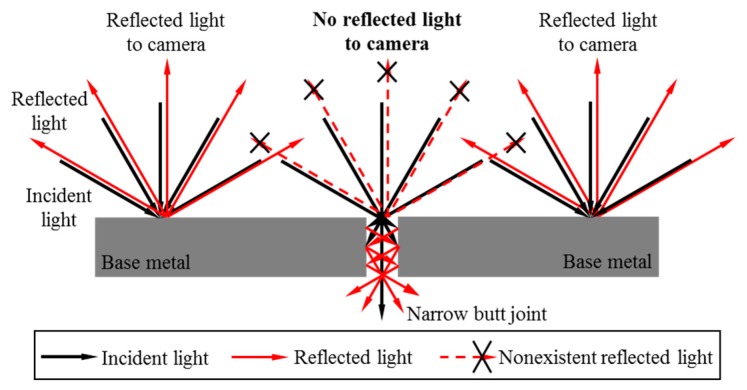
The reflection situation of the workpiece surface in uniform lighting condition.

Figure 6 shows the reflection situation of the workpiece surface in uniform lighting condition. Although there is no gap left purposely after assembly, an extremely small gap would exist due to the machining errors or slight morphological differences between the sidewalls of two workpieces. The gap may be less than 0.1 mm, but we exaggerate the gap in Figure 6 for better explanation. Due to the small gap, some of the incident light would passes through the narrow butt joint and some is reflected or absorbed by the sidewall of the groove, as shown in Figure 6. Therefore, the reflected light intensity in the narrow butt joint region is lower than the intensity in the base metal region. When the LSLS in our sensor is on, there is dark curve in the captured image, indicating the position of the narrow butt joint. In order to recognize the extremely small gap of the narrow butt joint, the image resolution of our sensor is designed to be about 12.5 μm at 30 mm distance as mentioned above.

**Figure 7:**
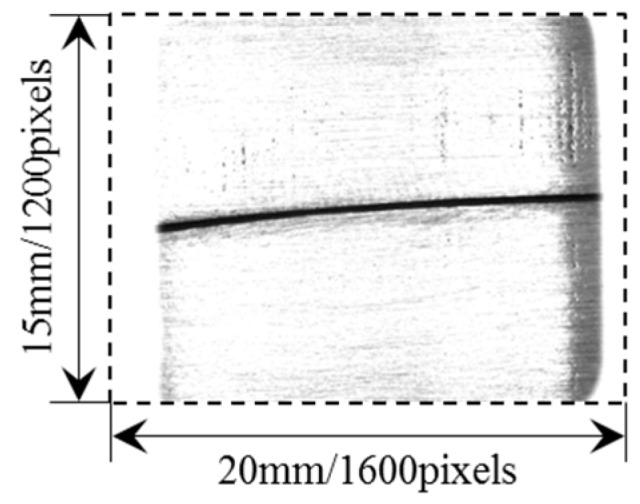
The grayscale image captured by the camera when the LSLS is on.

Figure 7 shows one of the images captured by the camera when the LSLS is on. The actual width of the narrow butt joint is about 0.1 mm. The grayscale of the base metal is nearly saturated and the grayscale of the narrow butt joint is nearly zero, which is beneficial to an accurate and fast groove detection.

According to the image captured above, we propose a corresponding processing algorithm to calculate the 2D pixel coordinates of the narrow butt joint. Considering that the FOV of our sensor is small (about 15 mm × 20 mm at 30 mm distance), the groove can be partially treated as a straight line approximately. The steps of our image processing algorithm are as follows:

1. Sub-image division. We divide the original image into several sub-images. In each sub-image, the groove is more like a straight line. The original image is divided into four sub-images along its column direction in this paper.
2. Valid pixel points extraction. Let the threshold *T* be 20% of the maximum grayscale in the image. Scan each column of the sub-image and find the longest line segment whose grayscale is less than *T*. Denote the center of the longest line segment in each column as the valid pixel point.
3. Hough transform [36]. We apply Hough transform to each sub-image and only the valid pixel points are considered when transforming. In Hough transform, each valid pixel point (*x*,*y*) in the image corresponds to a sine curve *ρ* = *x*cos *θ* + *y*sin *θ* in the parameter space (*ρ*,*θ*), where *ρ* and *θ* are the dependent and independent variables in the parameter space respectively. The Hough accumulator matrix *A*(*ρ*,*θ*) is obtained afterward. Since the groove is not exactly a straight line, the resolution of Hough accumulator should not be too small. We set the Hough accumulator resolution to Δ*ρ* = 10 and Δ*θ* = 5°.
4. Maximum accumulator selection. Find the maximum position *A*_max_ in the Hough accumulator matrix *A*(*ρ*,*θ*). The maximum accumulator *A*_max_ indicates the position of the groove curve. Traverse all of the valid pixel points and find the corresponding points whose mapped sine curves *ρ* = *x*cos *θ* + *y*sin *θ* pass through the maximum accumulator *A*_max_. These points are all located in the groove curve.
5. Linear interpolation. According to the points found in Step 4, we use the linear interpolation method to calculate the whole groove curve.

**Figure 8:**
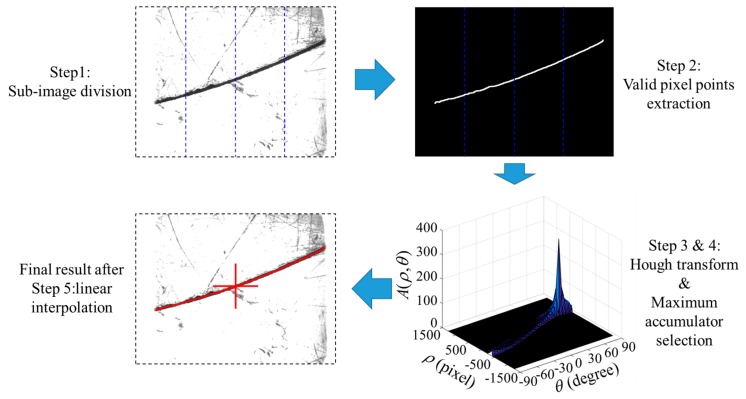
The output of each step in the proposed narrow butt joint detection algorithm.

Figure 8 shows the flow of our processing algorithm. The final detection result is marked in red in Figure 8. Our algorithm is used to process over 10,000 test images (different materials such as aluminum alloy, stainless steel, etc., different groove curves such as S-curve, straight line, polyline, etc., different surface roughness from 0.8 μm to 12.5 μm). The results show that the time cost of our proposed algorithm is less than 5 ms per frame; the detection error between the detection result and the actual groove position is not more than five pixels. It indicates that our proposed narrow butt joint detection method is suitable for real-time and accurate path teaching process.

Since our proposed algorithm uses Hough transform to detect the groove curve and Hough transform is not sensitive to the noises [36], it will not find a scratch instead of the joint as long as the length of the groove curve is longer than the length of the scratch.

## 5. Calculation of 3D Position and Normal Vector Coordinates of the Groove

**Figure 9:**
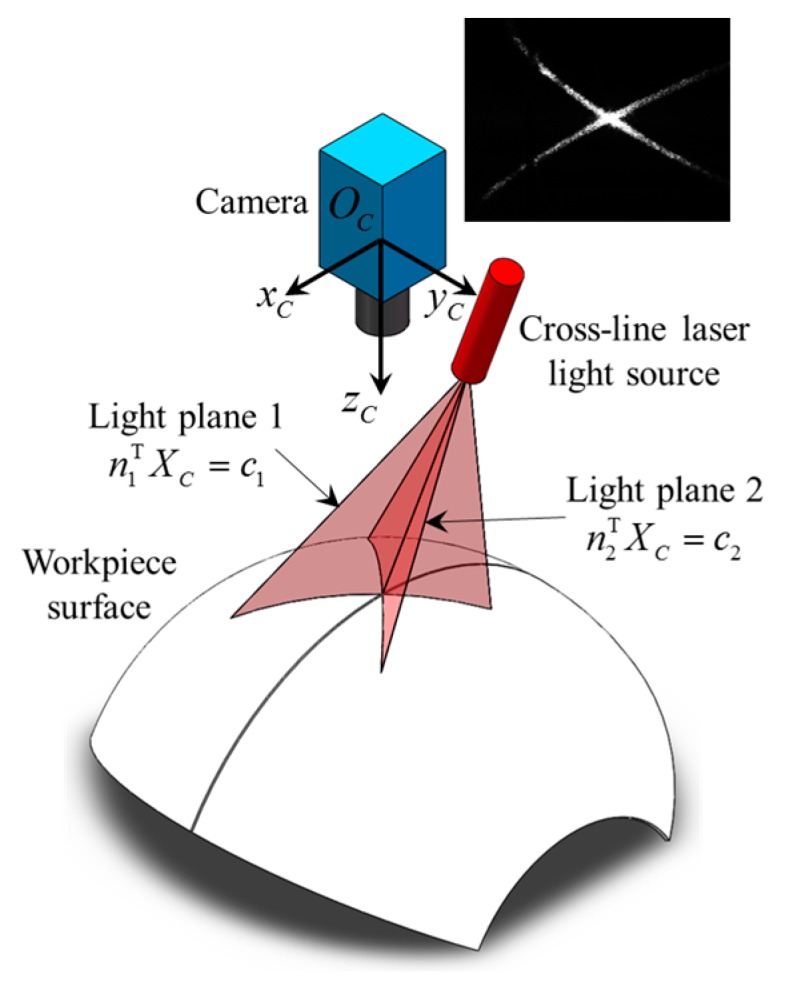
The principle of the cross-line laser method.

When the LSLS is on, we can only obtain the 2D pixel coordinates of the groove curve. The 3D information of the groove is still unknown. In this section, we use the cross-line laser stripes to calculate the 3D position and normal vector coordinates of the groove curve. Figure 9 shows the scene when CLLLS is on. The camera coordinate system {*C*} is established using Zhang’s method [34].

According to the pinhole model of the camera [34], the 2 × 1 pixel coordinate ***p*** in the image corresponds to any 3D point along a certain line. The equation of this line in camera coordinate system {*C*} is determined by,

(1) $$P_{C}=z_{C}\cdot S(p)=z_{C}\cdot \left[\begin{array}{c} f_{1}(p)\\ f_{2}(p)\\ 1 \end{array}\right],$$

where ***P****_C_* is a 3 × 1 vector representing any point on the line; *z_C_* is the third component of ***P****_C_*; and *f*_1_ and *f*_2_ are the mapping functions from pixel coordinate in the image to 3D coordinate in camera coordinate system {*C*}, which are determined by the intrinsic parameters of the camera [34]. The pixel coordinate ***p*** in the image is detected using Hough transform algorithm [36] in this paper, and the intrinsic parameters of the camera is calibrated by Zhang’s method [34].

The light plane equation of each laser stripe in Figure 9 can be calibrated beforehand using Zou’s method [35]. Suppose that the light plane equations of two laser stripes in camera coordinate system {*C*} are,

(2) $$n_{i}^{T}X_{C}=c_{i},\;\;i=1,2,$$

where ***X****_C_* represents the 3 × 1 coordinate of any point on the light plane in {*C*}, ***n****_i_* is the 3 × 1 unit normal vector of the light plane in {*C*}, and *c_i_* is the directed distance between the optical center of the camera (i.e., *O_C_* in Figure 9) and the light plane.

Suppose that ***q****_ij_* is 2 × 1 pixel coordinate of the *j*-th point in the *i*-th laser stripe. Combining Equations (1) and (2), the corresponding 3D coordinate ***Q****_ij_* (3 × 1 vector) in camera coordinate system {*C*} can be solved by,

(3) $$Q_{ij}=\frac{c_{i}}{n_{i}^{T}S(q_{ij})}S(q_{ij}).$$

If we move the workpiece at a suitable speed using our experiment platform, and the camera is controlled to be positioned exactly above the narrow butt joint (which will be discussed in Section 6), the 3D point cloud data of the workpiece surface can be obtained according to Equation (3). Suppose that there are *N* 3D points and their coordinates in camera coordinate system {*C*} are ***G***_1_, ***G***_2_, …, ***G****_N_* respectively. ***G***_1_, ***G***_2_, …, ***G****_N_* are all 3 × 1 vectors.

When the LSLS is on, we can obtain the 2D groove curve in the image. If ***γ*** is the 2 × 1 pixel coordinate of any point in the groove curve, then according to Equation (1), the 3D coordinate ***Γ*** (3 × 1 vector) in {*C*} corresponding to ***γ*** is located in the line,

(4) Γ=α⋅S(γ),

where *α* is an unknown parameter. Our goal is to calculate the intersection point ***Γ*** of the line determined by Equation (4) and the surface represented by 3D point cloud ***G***_1_, ***G***_2_, …, ***G****_N_*.

Supposing that the workpiece surface near the line determined by Equation (4) is approximately a flat plane, its equation in camera coordinate system {*C*} is,

(5) $$n^{T}K_{C}=c,$$

where ***K****_C_* represents the 3 × 1 coordinate of any point on the workpiece surface in {*C*}, ***n*** is the 3 × 1 unit normal vector of the workpiece surface near the line determined by Equation (4) in {*C*}, and *c* is the directed distance between the optical center of the camera and the workpiece surface. ***n*** and *c* are unknown parameters. The plane Equation (5) can be fitted using the points near the line determined by Equation (4). Once the plane Equation (5) is obtained, we can calculate the relative position and pose between the camera and the workpiece surface. Combining the calibrated transformation matrices between {*C*} and {*W*}, we can finally obtain the relative position and pose between the welding torch and the workpiece surface.

According to the analytic geometry theory, we can calculate the distance between ***G****_k_* and the line determined by Equation (4) as follows:

(6) $$d(G_{k})=\left\|G_{k}-\frac{S^{T}(\gamma )G_{k}}{S^{T}(\gamma )S(\gamma )}S(\gamma )\right\|,\;\;k=1,2,\cdots ,N,$$

and set a weight *w*(***G****_k_*) for each point ***G****_k_*. The weight *w*(***G****_k_*) decreases as *d*(***G****_k_*) increases, and once *d*(***G****_k_*) is larger than a certain threshold, *w*(***G****_k_*) decreases to zero rapidly. For example, the weight *w*(***G****_k_*) can be,

(7) $$w(G_{k})=\exp\left\{-\frac{{\left[d(G_{k})\right]}^{2}}{2\sigma^{2}}\right\},$$

where *σ* is a parameter which determines the correlation between ***G****_k_* and ***Γ***.

In this paper, we solve the equation of the workpiece surface by solving the following optimization problem:

(8) $$\left\{ \begin{array}{l} \underset{n,c}{\min}\;g(n,c)=\underset{n,c}{\min}\frac{1}{N}\sum_{k=1}^{N}w(G_{k}){\left|n^{T}G_{k}-c\right|}^{2}\\ s.t\;\;\left\|n\right\|=1 \end{array}\right.,$$

where the length of ***n*** is constrained to 1 to prevent overfitting. Equation (8) minimizes the weighted sum of the squared distances between ***G****_k_* and the plane determined by (5).

We use Lagrange multiplier method to solve Equation (8) as follows:

(9) $$h(n,c)=g(n,c)+\lambda (1-n^{T}n)=\frac{1}{N}\sum_{k=1}^{N}w(G_{k}){\left|n^{T}G_{k}-c\right|}^{2}+\lambda (1-n^{T}n),$$

where *λ* is the Lagrange multiplier.

Calculate the partial derivatives of Equation (9) and set them to 0 as follows:

(10) $$\left\{ \begin{array}{l} \frac{\partial h(n,c)}{\partial n}=2\left[\frac{1}{N}\sum_{k=1}^{N}w(G_{k})(G_{k}G_{k}^{T}n-cG_{k})-\lambda n\right]=0\\ \frac{\partial h(n,c)}{\partial c}=\frac{2}{N}\sum_{k=1}^{N}w(G_{k})(c-G_{k}^{T}n)=0 \end{array}\right..$$

After simplifying Equation (10), there is,

(11) $$\left\{ \begin{array}{l} \Omega \cdot n=\lambda \cdot n\\ c=n^{T}\frac{\sum_{k=1}^{N}w(G_{k})G_{k}}{\sum_{k=1}^{N}w(G_{k})} \end{array}\right.,$$

where ***Ω*** is a 3 × 3 matrix defined as,

(12) $$\Omega =\frac{\sum_{k=1}^{N}\sum_{s=1}^{N}w(G_{k})w(G_{s})(G_{k}-G_{s}){\left(G_{k}-G_{s}\right)}^{T}}{2N\sum_{k=1}^{N}w(G_{k})}.$$

Combining Equations (8), (11) and (12), there is,

(13) g(n,c)=λ.

According to Equations (8)–(13), the optimization problem (8) is solved only when the Lagrange multiplier *λ* is the minimum eigenvalue of the matrix ***Ω*** and the normal vector ***n*** is the eigenvector corresponding to *λ*. Since ***Ω*** is a symmetric matrix, we can calculate its minimum eigenvalue *λ* and the corresponding eigenvector ***n*** using Jacobian eigenvalue algorithm. Once the normal vector ***n*** is determined, the unknown parameter *c* can be solved by Equation (11). Therefore, we can obtain the plane equation ***n***^T^***K****_C_* = *c* near the line determined by Equation (4).

Combining Equations (4) and (5), the intersection point ***Γ*** of the workpiece surface and the line determined by Equation (4) can be calculated by,

(14) $$\Gamma =\frac{c}{n^{T}S(\gamma )}S(\gamma ).$$

The 3D position ***Γ*** and normal vector ***n*** of the groove can be determined by Equations (14) and (11) respectively. Thus, the 3D information of the narrow butt joint is obtained now.

## 6. 3D Path Teaching Experiments and Discussions

**Figure 10:**
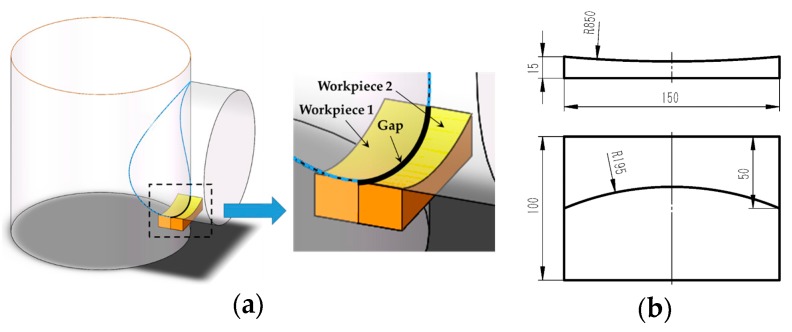
The workpiece sample used in this paper. (**a**) The intersection curve of two cylinders; (**b**) The CAD model of the workpiece sample. The unit of the dimensions in the figure is millimeter (mm).

Based on the research work in the previous sections, we carried out the 3D path teaching experiments using the platform shown in Figure 3. The workpiece with 3D narrow butt joint used in this paper is shown in Figure 10. It was made of stainless steel and the groove curve was the intersection curve of two cylinders.

**Figure 11:**
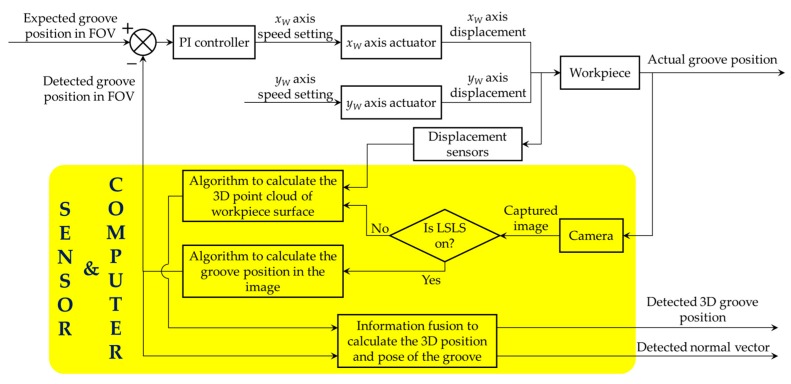
The block diagram representing the flow of the path teaching process.

During the path teaching process, the workpiece moves along the *y_W_* axis at a uniform speed. In the meantime, the camera generates the trigger signal and makes the LSLS and CLLLS switch on alternately. The industrial computer calculates the 3D position and normal vector coordinates of the groove based on the captured images. Therefore, the deviation between the groove and the center of FOV will be corrected using a PI controller. The output of the PI controller is the speed of *x_W_* axis. The proportional and integral gain of the PI controller were set to 100 and 0.01 respectively. The frame rate of the camera was set to 30 fps. The speed of *y_W_* axis was set to 2300 mm/min. In this way, the visual sensor recognizes the groove position automatically and the path teaching process is finished. The flow of the path teaching process is shown in the block diagram of Figure 11.

At any time *t*, suppose that ***P****_C_* (3 × 1 vector) and ***n****_C_* (3 × 1 vector) are the 3D position and normal vector coordinates respectively in camera coordinate system {*C*} detected by the visual sensor; *s_x_* and *s_y_* are the displacements of the *x_W_* and *y_W_* axes respectively, which can be obtained from the servo motors of the translational stage. We can calculate the corresponding 3D position ***P****_W_* (3 × 1 vector) and normal vector ***n****_W_* (3 × 1 vector) in world coordinate system {*W*} at time *t* = 0 by,

(15) $$\left\{ \begin{array}{l} P_{W}=R_{CW}\cdot P_{C}+T_{CW}-\left[\begin{array}{c} s_{x}\\ s_{y}\\ 0 \end{array}\right]\\ n_{W}=R_{CW}n_{C} \end{array}\right.,$$

where ***R****_CW_* and ***T****_CW_* are the 3 × 3 rotational and 3 × 1 translational transformation matrices from {*C*} to {*W*}. ***R****_CW_* and ***T****_CW_* can be determined beforehand by the common hand-eye calibration method.

**Figure 12:**
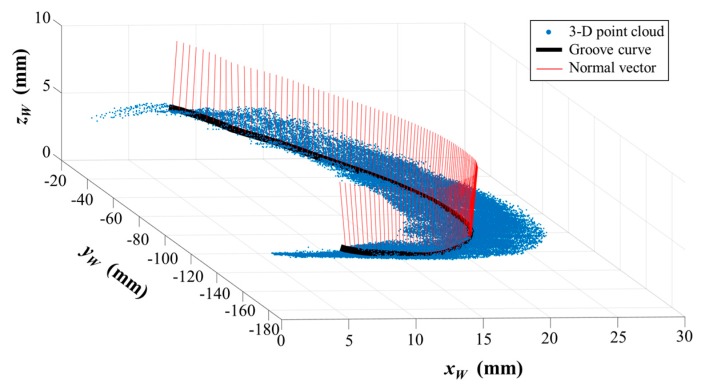
The 3D reconstruction result of the groove curve in one of the experiments.

According to Equation (15), the 3D position coordinate and normal vector of each point in the groove curve can be calculated in world coordinate system {*W*} after path teaching. Therefore, we obtain the 3D reconstruction results of the groove curve in world coordinate system {*W*}. Figure 12 shows the calculation result in one of our experiments. Another 100 experiments were conducted as well (different materials such as stainless steel and aluminum alloy, different surface roughness from 0.8 μm to 12.5 μm, and different relative position and pose between the welding torch and the workpiece). All the experiment results show that deviations between our detection results and the theoretical CAD model are not more than 0.24 mm and 0.54°. These errors may be caused by the machining errors and deformations of the workpiece. In addition, we used a coordinate measuring machine (CMM) with about 4 μm accuracy to measure the position of the actual groove curve. The position deviation between our detection result and the CMM result does not exceed 0.05 mm. It indicates that our method is suitable for narrow butt joint detection when there are machining errors and deformations.

**Figure 13:**
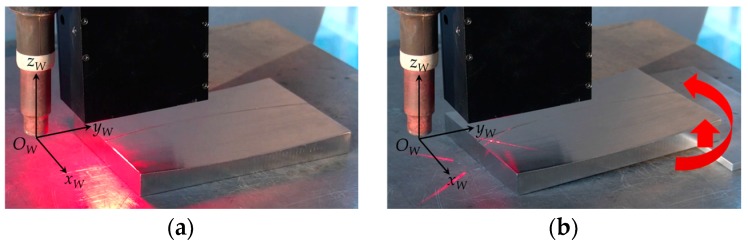
The path teaching experiment scenes when there are assembly errors. (**a**) The scene before movement and the LSLS is on; (**b**) The scene after movement and the CLLLS is on.

We also simulated the path teaching process when there are assembly errors, as shown in Figure 13. At the beginning, the workpiece was placed on the translational stage and we performed a path teaching process for the first time, as shown in Figure 13a. Then we lifted up one side of the workpiece using a 5 mm thick plate, and rotated the workpiece around *z_W_* axis, as shown in Figure 13b. Finally, path teaching was performed for the second time after movement.

**Figure 14:**
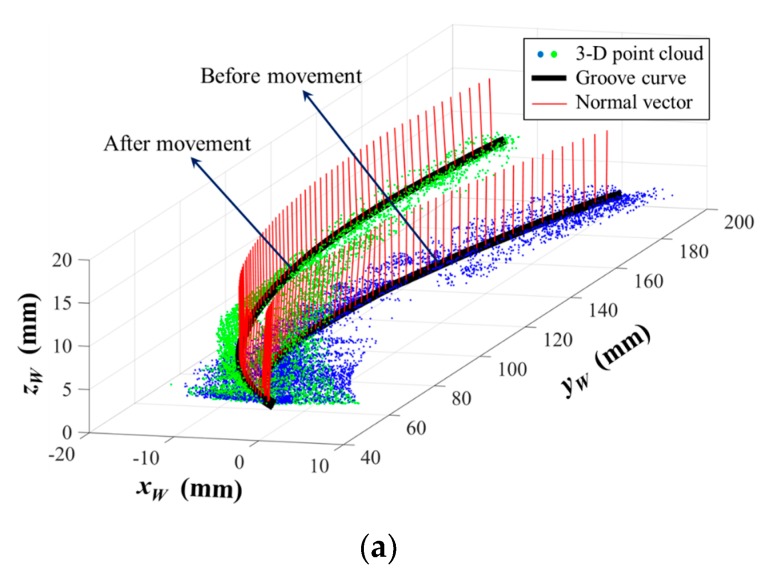

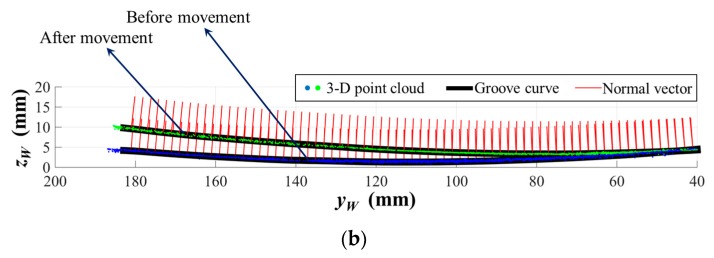
The 3D reconstruction results of the groove curve before movement and after movement. (**a**) Axonometric view of the reconstruction results; (**b**) Side view of the reconstruction results.

The 3D reconstruction results of the groove curve are shown in Figure 14. The 3D groove curves before and after movement were recognized accurately. The height difference along *z_W_* axis was about 5 mm between these two curves, which is exactly equal to the thickness of the lifted plate. The experiment results indicate that our proposed path teaching method is suitable for the situations where there are assembly errors before welding.

## 7. Conclusions

This paper proposes a vision-aided 3D path teaching method before narrow butt joint welding. Our designed visual sensor can capture images in different lighting conditions. When the LED surface light source is on, the grayscale of the narrow butt joint is quite different from the grayscale of the base metal. After image processing, the 2D pixel coordinates of any point in the groove curve can be obtained in less than 5 ms. When the cross-line laser light source is on, the laser stripe image can be captured by the visual sensor, and the 3D point cloud data of the workpiece surface can be obtained after calculation. We propose a synchronous acquisition method: two light sources are triggered to switch on alternately and the camera is synchronized to capture images when each light source is on. Compared with the traditional measurement methods using separate cameras, the proposed synchronous acquisition method only uses one camera, which eliminates the error source from calibration between two separate measurements. Different visual information can be fused to calculate the 3D position and normal vector coordinates of each point in the groove curve. 3D path teaching experiments were carried out to examine the applicability of our proposed method. Experiment results show that the image resolution can reach 12.5 μm at 30 mm working distance and that the proposed method is suitable for 3D narrow butt joint recognition before welding, which can compensate the machining errors, assembly errors, deformations, etc. of the workpiece. Our research can be applied to automatic path teaching in complex 3D components in aerospace and precision instrument industries. Further research will focus on the further development of our visual sensor (such as avoiding the interference from tack welds, more accurate control algorithm during path teaching, improving detection accuracy, etc.) and its applications in different welding methods (such as GTAW, laser welding, friction stir welding, etc.).
